# Iclaprim reduces the incidence and severity of *Staphylococcus aureus*-induced septic arthritis in a murine model

**DOI:** 10.1099/acmi.0.000052

**Published:** 2019-08-20

**Authors:** D. B. Huang, S. Noviello, C. G. Gemmell

**Affiliations:** ^1^ Motif BioSciences, Princeton, NJ, USA; ^2^ Rutgers New Jersey Medical School, Trenton, NJ, USA; ^3^ Division of Infection, Inflammation and Immunology, University of Glasgow Medical School, Glasgow, UK

**Keywords:** iclaprim, septic arthritis, murine model

## Abstract

*
Staphylococcus aureus* is the most common non-gonococcal aetiology of septic arthritis. The efficacy of iclaprim against *S. aureus* LS-1, a clinical strain identified from a patient with septic arthritis, was studied in MF1 mice to evaluate the activity of iclaprim, which is in clinical development, in preventing joint infections. Iclaprim (2.5–80 mg kg^−^^1^) administered as a single dose via the tail vein reduced the incidence of *S. aureus* septic arthritis and mortality in an experimental murine model of septic arthritis.

## Introduction

Septic arthritis is an orthopedic emergency leading to potential joint destruction and associated morbidity and mortality [1]. Septic arthritis occurs when micro-organisms infect the joint space by direct inoculation or by haematogenous spread. Direct introduction of micro-organisms into the joint space can result from procedures including intra-articular injection, joint aspiration and/or joint surgery. *Staphylococcus aureus* is the most common non-gonococcal aetiology, with over 25 % of isolates in the United States and in southern/eastern Europe having methicillin resistance (methicillin-resistant *S. aureus *, MRSA) [2, 3]. Because of the increasing resistance of MRSA to available antimicrobial agents, new ones that are efficacious and safe are needed to prevent and treat septic arthritis.

Iclaprim, which is in clinical development, is a selective bacterial dihydrofolate reductase inhibitor with potent activity against *S. aureus in vitro* and in clinical trials of acute bacterial skin and skin structure infections [4–6]. In a recent surveillance study of patients with skin infections in North America and Europe, iclaprim had an MIC_90_ of 0.12 µg ml^−1^ for 314 isolates of MRSA and one of 0.06 µg ml^−1^ for 304 isolates of methicillin-sensitive *S. aureus* [7].

A murine model was developed to evaluate iclaprim and its ability to prevent joint infections caused by *S. aureus* in mice. This is the first study of iclaprim activity in a murine model of *S. aureus *-induced septic arthritis. Iclaprim was studied because compared to trimethoprim [the only US Food and Drug Administration (FDA)-approved dihydrofolate reductase inhibitor], it has a lower MIC_90_ for *S. aureus *, can be given without a sulfonamide, overcomes select trimethoprim resistance and does not cause hyperkalemia, unlike trimethoprim/sulfamethoxazole, which is frequently used for the treatment of septic arthritis.

## Methods

All procedures in this study were in compliance with the Protection of Animals Act. This animal experiment complied with the National Institutes of Health guide for the care and use of laboratory animals [8] and the guidelines were followed after review and approval by the Institutional Animal Care and Use Committee at the University of Glasgow, Glasgow (protocol number 463208). Two experimental groups were studied for the activity of iclaprim in *S. aureus *-induced septic arthritis. The first experimental group included a lower infection inoculum of 5×10^7^ colony-forming units (c.f.u.) ml^−1^ and higher doses of iclaprim (20 and 80 mg kg^−1^), and followed the mice to day 8. The second experimental group studied included a higher infection inoculum of 1×10^8^ c.f.u. ml^−1^ and lower doses of iclaprim (2.5, 5, 10 and 20 mg kg^−1^), and followed the mice to day 12.

### Antimicrobial agents

Iclaprim powder (Motif BioSciences, Princeton, NJ, USA) was used in the *in vivo* studies after resuspension in sterile deionized water. The range of doses of iclaprim (2.5 to 80 mg kg^−^^1^) have been studied in toxicology studies and are within the no-observed-adverse-effect level range of iclaprim administration to mice (unpublished data). Therefore, this was the dose range studied for this study.

### Bacterial growth media

Trypicase soy agar (TSA) plates (BBL, Franklin Lakes, NJ, USA), brain heart infusion (BHI) broth (BBL, Franklin Lakes, NJ, USA) and Cytodex beads (Sigma-Aldrich, St Louis, MO, USA) were used. The standard Clinical and Laboratory Standards Institute (CLSI) broth microdilution technique [9] was used to determine the MIC, performed in triplicate, for the strains employed in these studies.

### Bacterial strains and bacteria culture

*S. aureus* strain LS-1, which was isolated from a patient with septic arthritis and causes septic arthritis in a New Zealand white mouse, as previously described, was supplied by Professor Curtis Gemmel [9–11]. This strain was sensitive to iclaprim (MIC 0.12 µg ml^−1^) and trimethoprim/sulfamethoxazole (MIC 2 µg ml^−1^) and resistant to oxacillin (MIC 4 µg ml^−1^). *S. aureus* LS-1 was grown on TSA plates at 37 °C in 5 % CO_2_. The bacterial inoculum was prepared by resuspending several colonies of an overnight streak culture in saline and adjusting a 1 : 10 dilution of the suspension to an OD of 0.1 at 625 nm. The adjusted suspension was prepared to a final standardized concentration of 5×10^7^ c.f.u. ml^−1^ for experiment 1 and 1×10^8^ c.f.u. ml^−1^ for experiment 2. Bacterial counts were performed to determine inoculum size. These inoculum sizes were used based on previous published data indicating that intravenous injection of 1×10^7^ c.f.u. ml^−^^1^ *S *. *aureus* LS-1 cells has been shown to induce septicaemia that lasts for 24–48 h with localization in the joints after approximately 4–5 days [9, 12]. Furthermore, bacterial arthritis occurs in a high proportion (approximately 75 %) of Swiss mice in this model [12].

### Animals

Adult male (4–6 weeks old) Swiss white mice (MF1) weighing approximately 25 g were acclimated for 5 days prior to the first study day. Animals had free access to food and water throughout the study.

### Infection studies

In both experimental groups (one with a lower infection inoculum and the second with a higher infection inoculum), 0.2 ml of the stock *S. aureus* LS-1 inoculum was injected by intravenous administration into the tail vein because these infection inocula have been shown to consistently result in septic arthritis in this animal infection model [10, 12]. Subsequently, iclaprim (2.5–80 mg kg^−^^1^, which is 1–2×MIC) or vehicle control was intravenously injected into the tail vein within 20–30 min. These single doses (2.5–80 mg kg^−^^1^) were used based on prior studies showing that these doses result in 1 and 2 log_10_ c.f.u. reductions at 24 h post-treatment in a mouse abscess model [13].

After dosing with iclaprim or vehicle control, the mice were then examined individually at regular intervals to day 8 in experiment 1 and day 12 in experiment 2 and scored for morbidity, mortality and development of septic arthritis. The assessment of severity of joint involvement of four joints per animal was scored on a scale of 1–3, with 1 point for mild swelling or erythema or both, 2 points for moderate swelling and erythema and 3 points for marked swelling and erythema and occasionally ankylosis, as previously published [10, 12]. These changes were assessed and scored for each limb and the mean score per animal group was taken.

### Statistical analysis

Within each experimental group (one with a lower infection inoculum and the second with a higher infection inoculum), mean values and standard deviations were calculated for each dose group. An analysis of variance (ANOVA) was performed to determine statistical differences for the incidence of affected joints, severity of joint involvement and mortality by day by dose group or control. A *P*-value of <0.05 was considered a statistically significant difference between the dose groups.

## Results

In experiment 1 (lower infection inoculum), iclaprim at 80 mg kg^−^^1^ reduced the incidence of septic arthritis (Table 1). By day 4, all (100 %; 10/10) of the infected control mice had at least one affected joint compared with 55 % (5/9) of mice that received 80 mg kg^−^^1^ of iclaprim. The decreases on day 4 in the percentage of joints affected in mice that received a single dose of 80 mg kg^−^^1^ iclaprim (13.9 %) or 20 mg kg^−^^1^ iclaprim (15.0 %) relative to infected controls (53.1 %) were statistically significant and continued to day 8. The severity of joint infections was numerically reduced in mice given high-dose iclaprim, with a mean severity score of 1.33 in animals treated with 80 mg kg^−^^1^ iclaprim compared with 1.73 and 1.68 in the low-dose iclaprim and control groups, respectively. The survival rates at day 8 were 8/10, 7/10 and 6/10 for mice given 80 mg kg^−^^1^ and 20 mg kg^−1^ iclaprim and the infected controls, respectively.

**Table 1.** Experiment 1: development of septic arthritis and mortality rates in mice infected with *S. aureus* LS-1

**Day**	**Parameter**	* S. aureus* LS-1-infected mice*
		**Control**	**20 mg kg^−1^ iclaprim**	***P*-value**	**80** mg kg^−1^ **iclaprim**	***P*-value**
Day 4	Joints affected, *n*/*n* %	17/32 (53.1)	6/40 (15.0)	<0.01	5/36 (13.9)	<0.01
	Severity score, mean	1.65	1.0		1.0	
	Mortality, *n*/*n*	2/10	0/10		1/10	
Day 6	Joints affected, *n*/*n* %	13/32 (40.6)	10/32 (31.3)	ns	6/36 (16.7)	<0.01
	Severity score, mean	1.92	1.5		1.36	
	Mortality, *n*/*n*	3/10	2/10		1/10	
Day 8	Joints affected, *n*/*n* %	16/28 (57.1)	15/32 (46.9)	ns	6/32 (18.8)	<0.01
	Severity score, mean	1.68	1.73		1.33	
	Mortality, *n*/*n*	4/10	3/10		2/10	

*The assessment of joints affected and he severity score excludes mice that expired prior to the day of examination.

NS=not statistically significant.

In experiment 2 (higher infection inoculum), groups of five mice were given 0, 2.5, 5, 10, or 20 mg kg^−1^ of iclaprim with improved survival in the iclaprim-treated groups compared to the control animals (Table 2). By day 7, 4/6 control animals had died, compared to 0/5 in the 20 mg kg^−1^ iclaprim group and 1–2/5 in the mid-dose groups. By the end of the 12-day observation period, the survival rate in the 10 and 20 mg kg^−1^ groups was 80 % compared to 33 % in the control group. In addition, there was a numerically lower incidence of septic arthritis among mice treated with iclaprim (10–19 % in treated mice compared with 29 % in the infected control) at day 3. By day 12, the incidence of septic arthritis and the severity scores in the affected joints were similar across the groups, but the analysis was confounded by higher mortality rates in the untreated and low-dose animals.

**Table 2.** Experiment 2: incidence and severity of joint sepsis and mortality in mice infected with *S. aureus* LS-1

**Day**	**Parameter**		* S. aureus* LS-1-infected mice*
		**Control**	**2.5 mg kg^−1^ iclaprim**	**5 mg kg^−1^ iclaprim**	**10 mg kg^−1^ iclaprim**	**20 mg kg^−1^ iclaprim**
Day 3	Incidence of joint sepsis, *n*/*n* %	7/24 (29.2)	3/16 (18.8)	1/20 (5.0)	2/20 (10.0)	2/20 (10.0)
	Severity score, mean	1.6	1.3	1.0	1.5	1.5
	Mortality, *n*/*n*	0/6	1/5	0/5	0/5	0/5
Day 5	Incidence of joint sepsis, *n*/*n* %	9/24 (37.5)	3/16 (18.8)	2/20 (10.0)	3/20 (15.0)	6/20 (30.0)
	Severity score, mean	1.6	1.3	1.0	1.3	1.2
	Mortality, *n*/*n*	1/6	1/5	0/5	0/5	0/5
Day 7	Incidence of joint sepsis, *n*/*n* %	4/12 (33.3)	1/12 (8.3)	4/12 (33.3)	2/16 (12.5)	5/20 (25.0)
	Severity score, mean	1.4	1.0	1.0	1.5	1.6
	Mortality, *n*/*n*	4/6	2/5	2/5	1/5	0/5
Day 9	Incidence of joint sepsis, *n*/*n* %	2/8 (25.0)	1/8 (12.5)	4/12 (33.3)	4/16 (25.0)	4/20 (20.0)
	Severity score, mean	1.3	1.0	1.7	1.5	1.8
	Mortality, *n*/*n*	4/6	3/5	2/5	1/5	0/5
Day 12	Incidence of joint sepsis, *n*/*n* %	2/8 (25.0)	1/8 (12.5)	1/12 (8.3)	4/16 (25.0)	4/16 (25.0)
	Severity score, mean	1.8	2.0	2.0	1.5	2.0
	Mortality, *n*/*n*	4/6	3/5	2/5	1/5	1/5

*The assessment of joints affected and the severity score excludes mice that expired prior to the day of examination.

## Discussion

The development of arthritis and its severity in this murine model of *S. aureus* arthritis have been described [10, 12]. The histopathological appearance of the joints of mice inoculated with strain LS-1 has been shown to resemble that seen in human septic arthritis [10]. The tissue tropism may be due to specific binding of *S. aureus* to bone-specific sialoprotein [14]. The *S. aureus* LS-1 strain also possesses a number of genes that govern the expression of soluble virulence factors (e.g. alpha and beta haemolysins), which are important for the pathogenesis of septic arthritis, and are likely responsible in the early onset of septic arthritis.

This is the first reported study to examine the efficacy of iclaprim in this murine model of septic arthritis. The development of septic arthritis and mortality among the control animals (animals that did not receive iclaprim) in both experiments 1 (lower infection inoculum) and 2 (higher infection inoculum) were consistent with those already published [10, 12]. Overall, iclaprim was efficacious in this *S. aureus* joint infection model. Iclaprim is a selective bacterial dihydrofolate reductase inhibitor and is bactericidal against and suppresses exotoxin production (Panton–Valentine leukocidin, alpha-haemolysin and toxic shock syndrome toxin 1) in *S. aureus* [15]. Although iclaprim given intravenously as a single dose did not cure infection, doses as low as 10 mg kg^−1^ were efficacious in reducing septic arthritis and improving mortality in infected animals. With the higher *S. aureus* inoculum and lower doses of iclaprim, iclaprim was unable to prevent joint infection completely for 12 days, although both the incidence and severity of infection were greater in the untreated mice compared with those given iclaprim. This may be partially explained by the difference in the pharmacokinetics of iclaprim when inoculating *S. aureus* followed by administration of iclaprim into the tail vein compared to studies of mice infected intraperitoneally with *S. aureus* with iclaprim administered through the mouse tail vein [16]. Importantly, iclaprim at doses of 10 and 20 mg kg^−1^ resulted in protection against *S. aureus-*mediated lethality in this experiment. Even in the small groups with the lower *S. aureus* inoculum and higher doses of iclaprim, there was evidence that 80 mg kg^−1^ iclaprim provides some protection, given the low rate of mortality and septic arthritis.

These pilot studies had several limitations, including small sample sizes. Only a single dose of antibacterial agent was given to the mice in both experiments, unlike in the clinical setting, where multiple doses would be administered. Therefore, any protective effect of iclaprim in these experiments is likely attributable to the acute lowering of the infective load of bacteria for as long as drug levels *in vivo* are greater than the minimal inhibitory concentration/minimal bactericidal concentration. Iclaprim is bactericidal with a rapid kill rate rather than bacteriostatic, consistent with its activity even in this acute setting [6]. No histopathological observation was conducted to confirm the severity of the arthritis in this study; however, prior studies utilizing this model have validated the scoring of joint involvement with histopathological analyses [10, 12]. Furthermore, the laboratory that performed this study was the same one that performed a prior study with the exact same experimental methodology as this animal infection model [10]. In addition, there is an increased concentration of thymidine in mice (100-fold greater than humans), which bypasses the dihydrofolate reductase enzyme, antagonizing drug activity and increasing the dose required to observe antibacterial effects in rodents; therefore, extrapolation from the doses used in this study to the clinical setting is challenging. Further studies are planned using a thymidine kinase-deficient *S. aureus *.

In conclusion, the results from these pilot experiments demonstrate that a single dose of iclaprim can reduce the incidence of septic arthritis and decrease mortality among animals infected with *S. aureus *.
